# High mercury accumulation in deep-ocean hadal sediments

**DOI:** 10.1038/s41598-021-90459-1

**Published:** 2021-05-26

**Authors:** Hamed Sanei, Peter M. Outridge, Kazumasa Oguri, Gary A. Stern, Bo Thamdrup, Frank Wenzhöfer, Feiyue Wang, Ronnie N. Glud

**Affiliations:** 1 Lithospheric Organic Carbon (LOC) Group, Department of Geoscience, Aarhus University, 8000 Aarhus C, Denmark; 2 Geological Survey of Canada, Natural Resources Canada, 601 Booth St, Ottawa, ON K1A 0E8 Canada; 3 Department of Environment and Geography, Center for Earth Observation Science, University of Manitoba, Winnipeg, MB R3T 2N2 Canada; 4 Research Institute for Global Change, Japan Agency for Marine-Earth Science and Technology (JAMSTEC), 2-15 Natsushima-cho, Yokosuka, Kanagawa 237-0061 Japan; 5 Department of Biology, University of Southern Denmark, HADAL and Nordcee, 5230 Odense M, Denmark; 6 HGF-MPG Group for Deep Sea Ecology and Technology, Alfred-Wegener-Institute Helmholtz-Center for Polar and Marine Research, 27570 Bremerhaven, Germany; 7 Max Planck Institute for Marine Microbiology, 28359 Bremen, Germany; 8 Department of Ocean and Environmental Science, Tokyo University of Marine Science and Technology, Tokyo, Japan; 9 Danish Institute for Advanced Study (DIAS), University of Southern Denmark, Fioniavej 34, 5230 Odense, Denmark

**Keywords:** Biogeochemistry, Environmental sciences, Ocean sciences, Marine chemistry

## Abstract

Ocean sediments are the largest sink for mercury (Hg) sequestration and hence an important part of the global Hg cycle^1^. Yet accepted global average Hg flux data for deep-ocean sediments (> 200 m depth) are not based on measurements on sediments but are inferred from sinking particulates^2^. Mercury fluxes have never been reported from the deepest zone, the hadal (> 6 km depth). Here we report the first measurements of Hg fluxes from two hadal trenches (Atacama and Kermadec) and adjacent abyssal areas (2–6 km). Mercury concentrations of up to 400 ng g^−1^ were the highest recorded in marine sediments remote from anthropogenic or hydrothermal sources. The two trench systems differed significantly in Hg concentrations and fluxes, but hadal and abyssal areas within each system did not. The relatively low recent mean flux at Kermadec was 6–15 times higher than the inferred deep-ocean average^1,3^, while the median flux across all cores was 22–56 times higher. Thus, some hadal and abyssal sediments are Hg accumulation hot-spots. The hadal zone comprises only ~ 1% of the deep-ocean area, yet a preliminary estimate based on sediment Hg and particulate organic carbon (POC) fluxes suggests total hadal Hg accumulation may be 12–30% of the estimate for the entire deep-ocean. The few abyssal data show equally high Hg fluxes near trench systems. These results highlight a need for further research into deep-ocean Hg fluxes to better constrain global Hg models.

## Introduction

The Minamata Convention on Mercury to reduce human emissions of this priority toxic metal was ratified and entered into force in 2017 (www.mercuryconvention.org)^1^ ^1–3^. Attention is now turning to how rapidly Hg may decrease in the biosphere^4,5^, ultimately via storage in one of the planetary sinks—soils and ocean sediments^6,7^.

Oceans are crucial in understanding the global cycling and fate of anthropogenic Hg because they contain more Hg than the upper organic layers of soils and the atmosphere combined, and marine sediments are probably the largest long-term repository^1,8^. The rate of Hg accumulation on continental shelves (excluding estuaries) is constrained by numerous field studies and modeling^3,9^, and is estimated at 120–200 t Hg a^−1^ ^1,3^. However, knowledge of Hg accumulation in deep-ocean sediments (91% of ocean area^10^) is limited. There are no measured Hg flux data from the deepest, hadal areas of the oceans. Consequently, global conceptual models and budgets used water column particulate Hg:POC data, scaled to global marine OC burial rates^2^, to infer deep-ocean Hg accumulation of 240–600 t a^−1^ ^1,3^, equivalent to an average flux of 0.7–1.8 µg.m^−2^  a^−1^ over an area of 330 × 10^6^ km^2^ ^10^. Even measurements of Hg concentrations in deep-ocean sediments are sparse compared to coastal sediments (see Refs.^11–17^ for Hg concentration data on bathyal and abyssal sites; and Refs.^16,18,19^ for hadal sites).

The current calculations of deep-ocean Hg accumulation assumed a constant water column Hg:POC of 0.6 µg Hg g POC^−1^ ^2,20^. However, measurements at many Pacific Ocean sites indicate that Hg:POC increases several-fold down to at least 500 m depth because sinking particles preferentially retain Hg relative to carbon during remineralization^21^. Also, measurements of the particulate:dissolved Hg partitioning co-efficient in the pelagic ocean show > 20 × higher values than models assume, possibly because Hg is “regeneratively scavenged” by decaying particulate organic matter^22^ (POM) similar to metals such as zinc^23^. Thus, Hg may penetrate deeper into the ocean at higher rates than models suggest. Given these discrepancies, and because deep-ocean sediments may be the largest oceanic Hg sink, there is a critical need to verify the inferred accumulation estimates with data based on sediment sampling.

**Figure 1:**
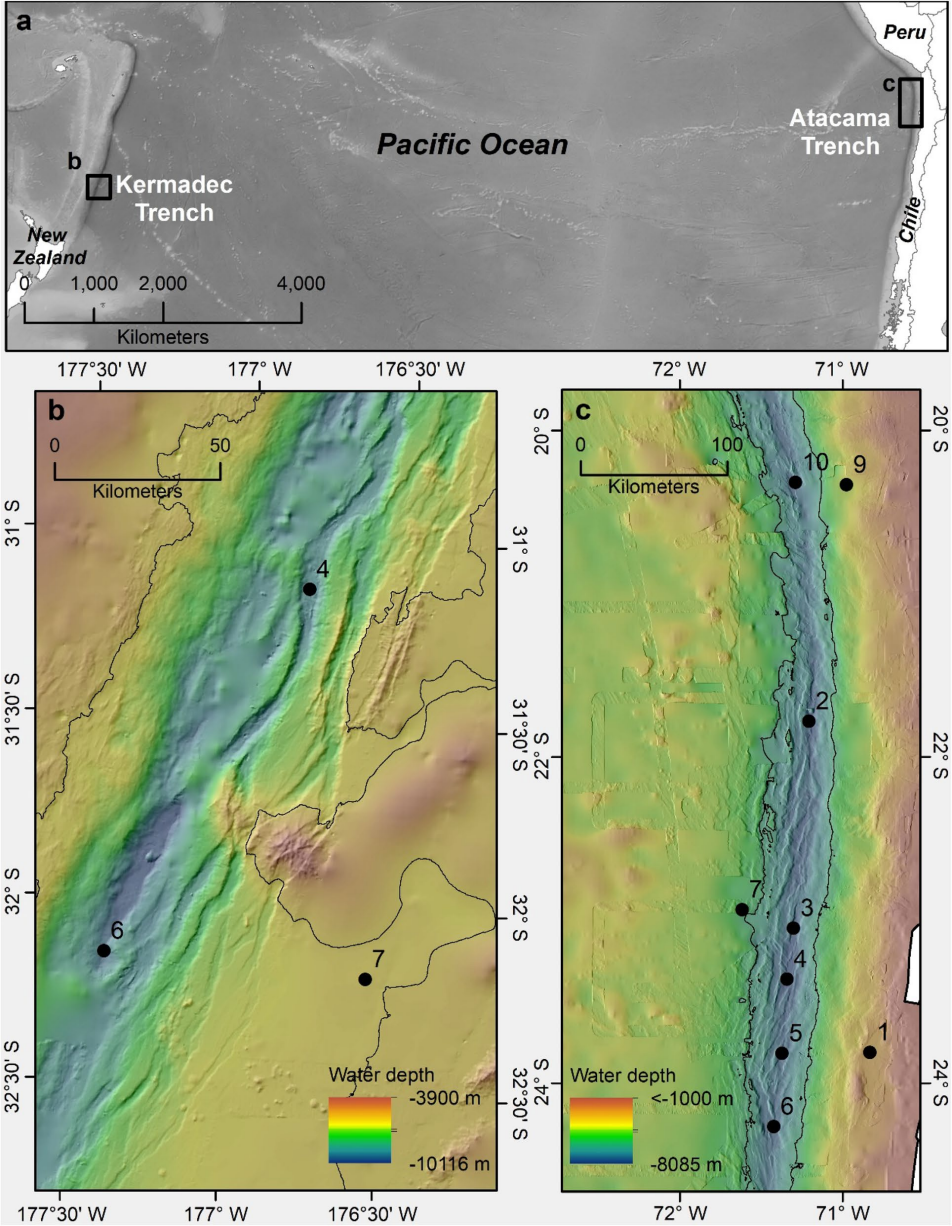
Study areas in the Kermadec Trench and the Atacama Trench regions in the Pacific Ocean (**a**). Detailed bathymetry data with specific sampling sites in the Kermadec Trench (**b**) and Atacama Trench (**c**). The sites were visited during two cruises with RV Tangaroa (TAN1711, 2017) and RV Sonne (SO261, 2018), respectively. Black line in (**b**) and (**c**) indicates the 6000 m depth contour. Black line in b and c indicates the 6000 m depth contour. Map adapted from Ref.^46^. Figure Copyright British Geological Survey UKRI 2021. Bathymetry data were obtained from the Global Multi-Resolution Topography Synthesis^47^.

Here we report Hg concentration and flux data from 12 sediment cores from two hadal subduction trenches, and adjacent abyssal plains and slope, in the Pacific Ocean (Atacama Trench, N = 9 sites, off Chile; Kermadec Trench, N = 3, near New Zealand; Fig. 1 and Table S1). There are 47 subduction trenches, troughs and trench faults in the world’s hadal zone, concentrated mainly in the Pacific^24^. Together, the Atacama and Kermadec trench systems represent 2.2% of the total hadal area of about 3.44 × 10^6^ km^2^ ^10,24^. Because they are the deepest parts of the ocean basin, trenches are natural accumulators of the organic and inorganic matter that was deposited at the ocean’s surface or produced in surface waters^25^. Atacama underlies one of the most productive oceanic regions and has the highest estimated POC flux of any trench (3.2 ± 1.4 g OC m^−2^ a^−1^), whereas Kermadec has a moderate POC flux (1.6 ± 0.5 g OC m^−2^  a^−1^) which is similar to the mean of 1.3 ± 0.8 g OC m^−2^  a^−1^ across 21 major subduction trenches > 6.5 km deep^26^.

## Results and discussion

Sediment dynamics in hadal trenches are governed by two processes: (1) continuous pelagic deposition and (2) infrequent mass wasting/deposition events that translocate large quantities of sediment from the trench slope to the trench interior (typically driven by seismic activity^27,28^). The profiles of sediment core excess ^210^Pb (^210^Pb_ex_: defined as the ^210^Pb fraction in secular disequilibrium from ^226^Ra daughters derived from the atmosphere and the water column) have clear indications of both processes (Fig. S1). Periods of monotonic exponential decreases of ^210^Pb_ex_ shown in most core sections reflect relatively constant sediment deposition. Sections of some Atacama cores (At 2, 3, 5, 7, 9 and 10) exhibit short-term ^210^Pb_ex_ trend reversals and plateaus that reflect dilution of ^210^Pb_ex_ by episodic mass deposition of low ^210^Pb_ex_ sediment. We calculated constant linear sedimentation rates (i.e., the sedimentation rates were unvarying down-core) from the periods of continuous deposition, excluding mass deposition events, and thereby provide a conservative estimate of overall sediment deposition rates at the trench axis. The Atacama cores exhibiting mass deposition events have similar calculated sedimentation rates to the other Atacama sites (Table S1).

For our purposes, we grouped Hg concentration data from the top 5–6 cm (4–5 slices; Fig. S2) of each core, where ^210^Pb_ex_ was present, as “recent” sediments which, based on the vertical accretion rates, represent material accumulated over about the last 60–190 years (median 120 years). The bottom five slices of each core, including sediments from 10 to 40 cm depth where ^210^Pb_ex_ was absent (see Fig. S1), were grouped as “historical” sediments. Although the historical samples can not be accurately dated, they occur at core depths well below the point of ^210^Pb_ex_ extinction, which would typically place their deposition prior to about the middle of the nineteenth century given ^210^Pb’s half-life of 22.3 years.

**Figure 2:**
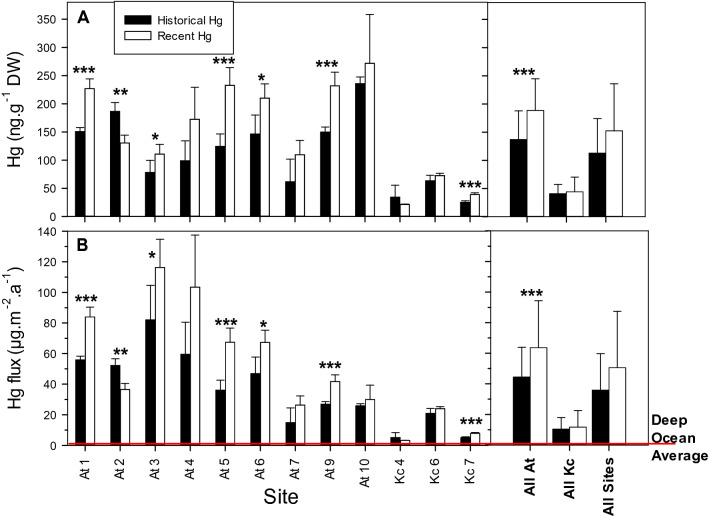
Plots of average (**A**) Hg concentrations and (**B**) Hg fluxes at the deep-ocean study sites. Left hand panels show concentration and fluxes for recent and historical sediments at individual sites. Right hand panels show concentrations and fluxes at all At and all Kc sites combined, and the concentrations and fluxes in recent and historical sediments of all sites. For individual sites, in (**A**) bars represent mean ± standard deviation (SD) Hg concentrations in the upper 5 cm (“recent”, N = 5) and bottom 5 slices (“historical”, N = 5) of sediment cores. In (**B**), bars represent mean ± SD Hg fluxes calculated from the mean and SD concentrations and sediment accumulation rates. Significant differences between recent and historical Hg concentrations and fluxes indicated by: *P < 0.05, **P < 0.01, ***P < 0.001. The Deep-Ocean Average line represents the inferred average flux over the entire deep-ocean based on Outridge et al*.*^1^ and Zhang et al*.*^3^.

Atacama sediments overall contained significantly higher Hg concentrations (P < 0.001) than Kermadec (Fig. 2). Sites in the trenches at both locations contained similar recent Hg concentrations to sites on the adjacent abyssal plains and slope (i.e., At1, At7, At9, and Kc7; the difference between trench and abyssal sites is non-significant at P > 0.05). Recent Atacama sediments on average had significantly higher Hg concentrations (188 ± 56 ng g^−1^; P < 0.001) than historical sediments (137 ± 51 ng g^−1^). The exception to this pattern was At2, in which recent sediments had lower Hg concentrations (P < 0.01) than historical. Recent Kermadec sediment Hg concentrations averaged across all sites (44 ± 26 ng g^−1^) were not significantly different from historical sediments (41 ± 16 ng g^−1^; P > 0.05), although they were higher at Kc7 (P < 0.001).

**Figure 3:**
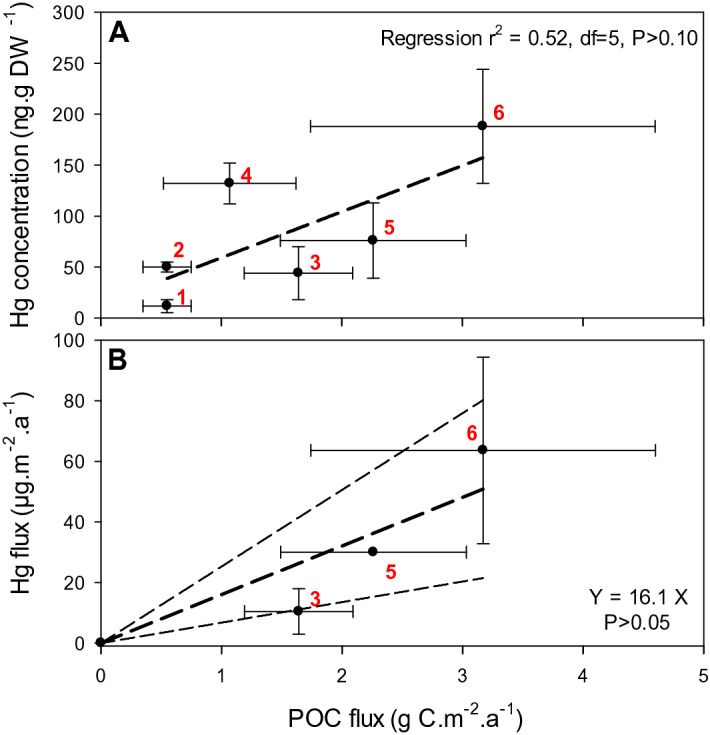
Plots of sediment Hg concentrations (**A**) and fluxes (**B**), and particulate organic carbon (POC) flux in hadal trench and abyssal plain sites world-wide. (Plots show mean ± SD values, for the top few cm of sediment cores or surface sediment grab samples. Study locations and Hg data sources in panel (**A**): 1—Marianna/Yap Trenches^18^, 2—Marianna Trench^19^, 3—Kermadec Trench and abyssal plain (this study), 4—New Britain Trench^19^, 5—Kuril-Kamchatka Trench and abyssal plain^16^, 6—Atacama Trench and abyssal plain and slope (this study). In panel (**B**), the locations and sources for data points 3 and 6 are the same as in panel A, but for point 5 the flux is from one core on the Kuril Basin abyssal plain^17^. Linear regression equations are shown as dashed lines, and in (**B**) the regression was forced through the origin. POC flux data are from Longhurst^26^ as tabulated by Stewart and Jamieson^24^ and Jamieson^35^.

The recent and historical Hg concentrations at Atacama were markedly higher than those in surface sediments in other oceanic regions remote from anthropogenic or hydrothermal sources, where Hg concentrations were usually < 80 ng g^−1^ ^9,15^. They were also higher than in other hadal and abyssal sites where mean concentrations ranged from 12 ± 6 to 132 ± 20 ng g^−1^ (Fig. 3A)^16,18,19^. The mean Atacama values were even higher than in some regions affected by anthropogenic activities. For example, the most contaminated deep-ocean site in the Mediterranean Basin exhibited a mean Hg concentration of 77 ng g^−1^ ^13^. Maximum concentrations in recent Atacama sediments, which ranged up to 401 ng g^−1^ (see Fig. S2) are the highest reported from remote locations anywhere including in the Kamchatka Trench and abyssal plains (158 ng g^−1^ ^16^) and bathyal areas of the Southern Ocean (87 ng g^−1^ ^15^). They approach the maxima reported from some of the most contaminated shelf sites world-wide including the Bohai Sea, China (574 ng g^−1^^29^) and the Laurentian Trough, Canada (520 ng g^−1^ ^11^).

POM scavenging and sedimentation of Hg from the euphotic zone, which is ultimately sourced from atmospheric deposition, is likely the main source of Hg in deep-ocean sediments. This is evidenced by Hg isotope analyses of hadal sediments^18^, ocean Hg-primary productivity modeling^3^, and geochemical associations between Hg and POM especially diatoms in particle traps and sediments^15,17,22^. An analysis of the few Hg concentration and flux data available for hadal and abyssal sediments showed positive though non-significant correlations between estimated POC flux and sediment Hg concentrations and fluxes (Fig. 3) which are also suggestive of an upper-ocean source of POM-associated Hg.

There are two other possible sources or processes that can contribute to high Hg concentrations in deep-ocean sediments. First, post-depositional redistribution of Hg could occur following the decomposition of Hg-containing POM, releasing dissolved Hg to porewater. Its subsequent diffusion and adsorption onto authigenic Fe and Mn hydroxides in oxic surface sediments can result in Hg enrichment^9,12^. Diagenetic redistribution of Hg is typically negligible in lake and coastal sediments^11,30^, but is less studied and could be important in ocean basins due to different sedimentation and mixing regimes^12,13^. As porewater Hg concentrations were not measured in this study, we cannot directly estimate the magnitude of redistribution. Significant correlations between concentrations of solid phase Hg and reactive Fe (see Fig. S3), an association suggestive of Hg redistribution^12^, were observed in four of nine Atacama cores (At1, At5, At7, At9; Table S2). To test the effect of possible redistribution, we compared mean recent Hg concentrations with those in the whole cores. Whole core data represent the integrated averages of increased surface sediment concentrations and decreased lower core concentrations due to possible redistribution. Recent sediment Hg concentrations were only 16.7% higher on average than whole core concentrations at Atacama (188 vs 162 ng g^−1^), and 1.9% higher at Kermadec (44.3 vs 43.5 ng g^−1^). Higher Hg values in recent Atacama sediments but not in Kermadec could also be the result of elevated deposition of anthropogenic atmospheric Hg into the eastern Pacific Ocean, compared to lower levels of anthropogenic Hg in ocean waters near Kermadec^3^. While we cannot resolve the two alternate explanations, the absence of significant Hg-reactive Fe correlations at the majority of Atacama sites, and the small increase in recent Hg concentrations relative to whole cores at both trenches, suggests that any effect of redistribution on recent sediment Hg concentrations and fluxes was small.

A second possible Hg source in deep-ocean sediments is seafloor hydrothermal vents associated with mid-ocean ridges. Globally, vents are thought to contribute minor amounts of Hg to oceans, ≤ 100 t a^−1^ ^1,22^, but could be locally important. The Atacama study area is several thousand km east of the nearest known hydrothermal field^31^ indicating that, in this area with the highest Hg concentrations, hydrothermal influences are negligible. The Kermadec trench is located relatively close to a hydrothermal field^31^, but no studies have been conducted on vent releases or seawater Hg concentrations in this region. However, there is little evidence of significant hydrothermal impacts on Hg concentrations in seawater^32–34^ or biota^18^ in the vicinity of vent plumes elsewhere, suggesting that the possibility is unlikely in this study. Overall, therefore, the Hg concentrations in these trench sediments are likely to predominantly reflect POM-associated transport of Hg from the euphotic zone.

Mean total Hg fluxes in recent sediments at Atacama (63.6 ± 30.8 µg m^−2^ a^−1^) were significantly higher (P < 0.001) than in historical sediments (44.4 ± 19.5 µg m^−2^ a^−1^), and also significantly higher (P < 0.001) than recent Kermadec sediments (10.4 ± 7.5 µg m^−2^ a^−1^; see Fig. 2B). There was no significant difference between average fluxes measured in trenches and in abyssal plain and slope sites at either location (P > 0.10). Combined mean recent and historical Hg fluxes were 50.6 ± 36.8 (median 39.1) µg m^−2^ a^−1^ and 35.9 ± 23.8 (median 31.5) µg m^−2^ a^−1^, respectively. A similar range of fluxes (30–44 µg m^−2^ a^−1^) was reported in post-1900 sediments in a dated core from the abyssal Kuril Basin^17^, supporting these findings.

To place these results in context, the relatively low recent Hg flux measured at Kermadec (10.4 µg m^−2^ a^−1^) is 6–15 times higher than the currently estimated deep-ocean average of 0.7–1.8 µg m^−2^ a^−1^ ^1,3^. Because the POC flux at Kermadec is similar to the average of all of the world’s trenches > 6.5 km depth^26^, Kermadec may approximate the global average Hg flux in the hadal. The median recent flux across our study sites (39.1 µg m^−2^ a^−1^) was 22–56 times larger than the inferred deep-ocean average Hg flux, but the median includes Atacama with the highest POC flux in the world, and which therefore may have a substantially higher Hg flux than most other trenches. Overall, however, these results indicate that some hadal trenches and adjacent abyssal areas are intense focal points of Hg accumulation. The question arises as to what impact these high hadal fluxes might have on global deep-ocean Hg accumulation rates.

To tentatively calculate the total hadal zone Hg flux, the few available data from hadal and abyssal sites (the present study, and Aksentov and Sattarova^17^) were used to approximate Hg fluxes for each trench system using the much larger global trench POC database. Individual trench estimates were summed to arrive at a total hadal Hg accumulation rate. POC flux data are available for 18 of the deepest trenches > 6.5 km depth, including Atacama and Kermadec^24,35^, representing 92% of the global hadal area below 6.5 km. Applying a Hg:POC regression slope (± standard error, SE) of 16.1 ± 2.9 μg Hg g POC^−1^ (see Fig. 3B), we arrive at a preliminary estimate of total Hg burial of 16.9 ± 1.0 t a^–1^ in this zone (see Table S3). Scaling up to the much larger hadal area bounded by the 6 km isobath^10^, the total Hg flux is estimated to be 73 ± 4.4 t a^–1^. The error terms for the 18 trenches and for the total hadal zone were propagated from the SE of the Hg:POC regression slope applied to each trench’s POC data. We emphasize the uncertainty associated with the 73 t a^−1^ value because of the few hadal or abyssal sites in total that have been sampled for Hg fluxes (N = 3), the large variation in fluxes between cores within the study trenches, and the relatively large confidence intervals associated with the Hg:POC regression (see Fig. 3B).

## Conclusions

This study has substantially expanded the knowledge base concerning Hg flux in the deep-ocean. These results increase by six-fold the number of dated cores analyzed from this zone and provide the first estimates of hadal Hg flux, which is shown to be high at both study sites relative to the currently accepted deep-ocean average flux. Although still limited in number and geographical scope, the results can be placed in the context of the current estimate of deep-ocean Hg accumulation. Our first order estimate of 73 t a^−1^ for hadal Hg burial represents 12–30% of the putative deep-ocean accumulation of 240–600 t a^−1^ ^1,3^, occurring within an area equal to 1.1% of the total deep-ocean. This estimate omits Hg flux in the much larger abyssal zone which is 85% of total ocean area^10^. Given the evidence of similarly high Hg fluxes in some abyssal sediments (this study and Ref.^17^), and even higher fluxes in high productivity bathyal areas of the Great Southern Ocean^15^, it is possible that the total deep-ocean Hg accumulation is higher than is presently believed. Our results thus call for extensive additional sampling of the deep-ocean to better constrain these preliminary estimates and to improve global Hg modeling.

## Methods

### Sampling

Sediment was for most part recovered by a multi-corer^36^, but in some cases we were successful using box coring (site Kc7) or an autonomous lander system for core recovery (Kc4). Recovered sediment cores were transferred to an onboard laboratory that maintained in situ bottom water temperature (2–4 °C). For the Kermadec Trench system, sediment cores were sectioned in 1 cm slices down to 2 cm depth, 2 cm slices to 10 cm and subsequently in 5 cm slices to the core bottom. For the Atacama Trench system, the cores were sectioned in 1 cm slices down to 10 cm, 2.5 cm slices to 20 cm, followed by 5 cm slices until the bottom of the core. Samples were homogenized in plastic bags and stored at -20 °C until laboratory analysis onshore.

### Excess ^210^Pb (^210^Pb_ex_) analysis and sedimentation rate calculation

The procedures for ^210^Pb_ex_ analysis and sediment accumulation rate calculation are well established, and here followed previous studies on hadal sediments^37–39^. Sediment was dried in an oven at 80 °C for 48 h, then milled with a mortar. 2.0 g of sediment powder were transferred in a plastic tube and hermetically sealed. The tubes were left for more than 2 months to establish secular equilibrium between ^226^Ra and ^222^Rn. To obtain ^210^Pb_ex_ activity, counting peaks of the specific gamma rays from ^210^Pb (46.5 keV) and ^214^Pb (351.9 keV) were measured with well-type HPGe detectors (GWL120230 and GWL120-15, ORTEC) connected to a multichannel spectrum analyzer (APV8002, TechnoAP or MCA-7a, SEIKO EG&G). Counting peak areas of the target nuclides were calculated with Gaussian curve fitting with KaleidaGraph 4.5 (Synergy Software). To quantify these activities, Reference Uranium–Thorium ore DL-1a (CANMET) was used as the standard material. ^210^Pb_ex_ activity was calculated by subtracting ^214^Pb from the ^210^Pb based on an assumption of secular equilibrium between ^226^Ra and the daughter nuclei in the sediment. Sediment accumulation rates were calculated based on the Constant Initial Concentration model^40,41^ from the ^210^Pb_ex_ activity (Bq g^−1^) in the core sections displaying exponential decreases in the plots. Instead of the core depth (cm), cumulative mass depth (g cm^−2^) obtained from the core depth and the dry bulk density (g cm^−3^) was used to express the sediment accumulation rate (initially as g cm^−2^ a^−1^).

### Hg analysis

Mercury in the sediment samples was determined as total Hg on a Hydra IIC direct mercury analyzer (Teledyne Leeman Labs, Hudson, NH, USA) using thermal decomposition, amalgamation and atomic absorption spectrophotometry following EPA Method 7473^42^. 10–50 mg of individual freeze-dried sediment samples were weighed into quartz boats. Mercury concentrations were quantified by the external standard method with calibration curves ranging from 1 to 1500 μg g^−1^. Within every batch of 10 samples, 3 blanks, 1–2 pairs of duplicates and 2–3 certified reference materials (CRMs: MESS-3, DOLT-5; and NIST 2709a and PACS-3, obtained from the National Research Council of Canada and NIST, respectively) were analyzed. In order to be deemed valid, the CRMs must fall within the range of the certified mean ± standard deviation. If CRM concentrations were outside the acceptable range, 2 more CRMs were analyzed. If the following CRMs were still outside the acceptable range, the instrument was re-calibrated. The mean CRM recoveries were 101.9 ± 9.0% and were within the expected ranges.

### Reactive Fe analysis

Two reactive Fe extraction procedures were employed on frozen sediments—Fe-DCA (Fe species solubilized in a dithionite-citrate-acetic acid solution (50 mg mL^−1^ sodium dithionite in 0.35 M sodium citrate, 0.2 M acetic acid, pH 4.8 for 1 h)), and Fe-HCl (Fe species solubilized by a mild HCl extraction (0.5 M HCl for 1 h)). Fe-DCA extracts the non-silicate-bound Fe fraction (oxides and authigenic Fe(II) phases excluding pyrite), whereas Fe-HCl releases poorly crystalline oxides and authigenic Fe(II) but also leaches some Fe from silicates^43,44^. Fe in the extract solutions was determined as total Fe using Ferrozine^45^. Concentrations were calculated on a dry weight basis using the water content.

### Statistics

Concentration and flux data in the text are represented as mean ± standard deviation (SD) values. Mercury concentration and flux data were not normally distributed in some cores, and differences between recent and historical sediments in these cases were tested with a Mann–Whitney Rank sum test. Those with data that were normally distributed and which satisfied the assumption of equal variance were analysed with t-tests. Linear regressions analysed relationships between mean Hg concentrations and fluxes and POC fluxes because the combined data were normally distributed. We used SigmaPlot 14.0 Software (Systat Software Inc.). Error propagation was calculated with the online calculator at www.julianibus.de.

## Supplementary Information

Supplementary Information.

## Data Availability

The data that support the findings of this study are available from the corresponding author upon reasonable request.
