# Supplementary material for: High mercury accumulation in deep-ocean hadal sediments
Source: Sci Rep. 2021 May 26;11:10970. doi: 10.1038/s41598-021-90459-1 (PMC8155115; doi:10.1038/s41598-021-90459-1)
Supplement: Supplementary file 1 — Supplementary Information. [file 41598_2021_90459_MOESM1_ESM.docx]

**Supplementary Information**

**High mercury accumulation in deep-ocean hadal sediments**

by

Hamed Sanei^1^, Peter M. Outridge^1,2,3^*, Kazumasa Oguri^4,5^, Gary A. Stern^3^, Bo Thamdrup^5^, Frank Wenzhöfer^5,6,7^, Feiyue Wang^3^, and Ronnie N. Glud^5,8,9^

1 – Lithospheric Organic Carbon (LOC) Group, Department of Geoscience, Aarhus University, 8000 Aarhus C, Denmark.

2 – Geological Survey of Canada, Natural Resources Canada, 601 Booth St., Ottawa, Ontario K1A 0E8, Canada.

3 – Center for Earth Observation Science and Department of Environment and Geography, University of Manitoba, Winnipeg, Manitoba R3T 2N2, Canada.

4 – Research Institute for Global Change, Japan Agency for Marine–Earth Science and Technology (JAMSTEC), 2-15 Natsushima-cho, Yokosuka, Kanagawa, 237-0061, Japan.

5 – University of Southern Denmark, HADAL and Nordcee, Department of Biology, 5230 Odense M, Denmark.

6 – HGF-MPG Group for Deep Sea Ecology and Technology, Alfred-Wegener-Institute Helmholtz-Center for Polar and Marine Research, 27570 Bremerhaven, Germany.

7 – Max Planck Institute for Marine Microbiology, 28359 Bremen, Germany.

8 – Department of Ocean and Environmental Science, Tokyo University of Marine Science and Technology, Japan.

9 – Danish Institute for Advanced Study (DIAS), University of Southern Denmark, Fioniavej 34, 5230 Odense, Denmark

* Corresponding author: P.M. Outridge. Office: +1 613-996-3958. Email: peter.outridge@canada.ca”

CONTENTS

List of Figures

Fig. S1. Excess ^210^Pb activity plots in Atacama and Kermadec trench sediments.

Fig. S2. Mercury concentration profiles in Atacama (At) and Kermadec (Kc) sediments.

Fig. S3. Solid phase reactive Fe species profiles in Atacama sediments.

List of Tables

Table S1. Latitude and longitude co-ordinates, water depths, core lengths and calculated sediment accumulation rates for the 12 sediment cores retrieved at Atacama and Kermadec.

Table S2. Results of simple linear regressions between Hg and operationally-defined reactive Fe species in Atacama sediments.

Table S3. Calculation of Hg burial flux in global hadal sediments from POC flux.

Additional Reference

48. Fujiwara T., *et al.* Morphology and tectonics of the Yap Trench. *Marine Geophysical Research*, **21**, 69–86 (2000).

**Fig. S1.** Excess ^210^Pb activity plots in Atacama and Kermadec trench system sediments. (Only activity data which were above instrument detection limits are plotted).

**Fig. S2.** Mercury concentration profiles in Atacama (At) and Kermadec (Kc) sediments.

**Fig. S3**. Solid phase reactive Fe species profiles in Atacama sediments.

**Table S1.** Latitude and longitude co-ordinates, water depths, core lengths and calculated sediment accumulation rates for the 12 sediment cores retrieved at Atacama (At) and Kermadec (Kc). Zones are as described by Harris et al.^10^.

| Site | Zone Description | Latitude  (S) | | Longitude  (W) | | Water depth  (m) | | Core length  (cm) | Sediment accumulation rate  (g.m^-2^.a^-1^; mm.a^-1^)  (g.m^-2^.a^-1^; mm.a^-1^) | | |
| --- | --- | --- | --- | --- | --- | --- | --- | --- | --- | --- | --- |
|  |  |  |  | |  | |  | | |  |  |
| At1 | Slope | 23°48.72' | | 70°50.04' | | 2560 | | 20 | 370; 0.77 | | |
| At2 | Hadal | 21°46.86' | | 71°12.48' | | 7995 | | 25 | 280; 0.29 | | |
| At3 | Hadal | 23°02.94' | | 71°18.12' | | 7915 | | 30 | 1050; 0.85 | | |
| At4 | Hadal | 23°21.78' | | 71°20.60' | | 8085 | | 30 | 600; 0.49 | | |
| At5 | Hadal | 23°49.02' | | 71°22.32' | | 7770 | | 30 | 290; 0.42 | | |
| At6 | Hadal | 24°15.96' | | 71°25.38' | | 7720 | | 32 | 320; 0.44 | | |
| At7 | Abyssal | 22°56.22' | | 71°37.08' | | 5500 | | 25 | 240; 0.65 | | |
| At9 | Abyssal | 20°19.97' | | 70°58.70' | | 4050 | | 17.5 | 180; 0.33 | | |
| At10 | Hadal | 20°19.14' | | 71°17.46' | | 7770 | | 35 | 110; 0.26 | | |
| Kc4 | Hadal | 31°08.41′ | | 176°48.48′ | | 9300 | | 30 | 150; 0.30 | | |
| Kc6 | Hadal | 32°08.93′ | | 177°23.91′ | | 9555 | | 40 | 330; 0.42 | | |
| Kc7 | Abyssal ababyssalabyssal | 32°11.22′ | | 176°33.66′ | | 6080 | | 30 | 200; 0.30 | | |
|  |  |  |  | |  | |  | | |  |  |

**Table S2.** Results of simple linear regressions between concentrations of Hg and operationally-defined reactive Fe species in Atacama sediments.

See Methods for description of the operationally-defined species. All datasets passed normality and constant variance tests except for At 10 variance. In that case a Spearman Rank Correlation test gave the same non-significant result. * = P<0.05; ** = P<0.01. Kermadec sediments were not subjected to reactive Fe analysis).

| Site | Hg : Fe-DCA  r^2^ (total df; P value) | Hg : Fe-HCl  r^2^ (total df; P value) |
| --- | --- | --- |
| At 1 | 0.50 (7; 0.049*) | 0.38 (8; 0.101) |
| At 2 | 0.17 (9; 0.241) | 0.07 (10; 0.462) |
| At 3 | 0.27 (8; 0.154) | 0.39 (9; 0.075) |
| At 4 | 0.02 (10; 0.643) | 0.26 (11; 0.107) |
| At 5 | 0.50 (10; 0.016*) | 0.16 (11; 0.219) |
| At 6 | 0.21 (8; 0.214) | 0.10 (9; 0.419) |
| At 7 | 0.36 (9; 0.065) | 0.42* (10; 0.044) |
| At 9 | 0.58 (7; 0.028*) | 0.79** (8; 0.003) |
| At 10 | 0.13 (11; 0.253) | 0.00 (12; 0.931) |

**Table S3.** Calculation of total Hg burial flux in global hadal sediments from POC flux

|  | **Max Depth^a^** | **Area^a^** | **Mean POC flux^a^** | **Total POC flux^b^** | **Mean Hg flux^c^** | **Std. Error**  **Hg flux** | **Total Trench Hg flux^c^** | **Std. Error**  **Total Hg flux** | |  |
| --- | --- | --- | --- | --- | --- | --- | --- | --- | --- | --- |
|  | **m** | **km^2^** | **g C m^−2^ a^−1^** | **10^6^ kg a^–1^** | **μg Hg m^–2^ a^–1^** | **μg Hg m^–2^ a^–1^** | **t a^–1^** | **t a^–1^** | |  |
| ***Individual trenches (>6500 m)*** | | | | | | | | |  | |
| Aleutian | 7669 | 63036 | 1.76 | 110.9 | 28.3 | 5.1 | 1.8 | 0.3 | |  |
| Atacama | 7999 | 26415 | 3.17 | 83.7 | 51.0 | 9.2 | 1.3 | 0.2 | |  |
| Banda | 7329 | 9963 | 1.6 | 15.9 | 25.8 | 4.6 | 0.3 | 0.05 | |  |
| Izu-Bonin | 9701 | 99801 | 1.69 | 168.7 | 27.2 | 4.9 | 2.7 | 0.5 | |  |
| Java | 7204 | 22689 | 1.06 | 24.1 | 17.1 | 3.1 | 0.3 | 0.1 | |  |
| Kermadec | 10177 | 49733 | 1.64 | 81.6 | 26.4 | 4.8 | 1.3 | 0.2 | |  |
| Kuril-Kamchatka | 10542 | 91692 | 2.26 | 207.2 | 36.4 | 6.6 | 3.3 | 0.6 | |  |
| Mariana | 10920 | 79956 | 0.55 | 44.0 | 8.9 | 1.6 | 0.7 | 0.1 | |  |
| New Britain | 8844 | 11135 | 1.07 | 11.9 | 17.2 | 3.1 | 0.2 | 0.03 | |  |
| New Hebrides | 7156 | 2439 | 0.86 | 2.1 | 13.8 | 2.5 | 0.03 | 0.01 | |  |
| Palau | 8021 | 1692 | 0.61 | 1.0 | 9.8 | 1.8 | 0.02 | 0.00 | |  |
| Philippine | 10540 | 54198 | 0.69 | 37.4 | 11.1 | 2.0 | 0.6 | 0.1 | |  |
| Puerto-Rico | 8526 | 52992 | 0.85 | 45.0 | 13.7 | 2.5 | 0.7 | 0.1 | |  |
| Ryukyu | 7531 | 14652 | 0.9 | 13.2 | 14.5 | 2.6 | 0.2 | 0.04 | |  |
| San Cristobal | 8641 | 9576 | 0.82 | 7.9 | 13.2 | 2.4 | 0.1 | 0.02 | |  |
| South Sandwich | 8125 | 31293 | 0.66 | 20.7 | 10.6 | 1.9 | 0.3 | 0.1 | |  |
| Tonga | 10800 | 65817 | 0.99 | 65.2 | 15.9 | 2.9 | 1.0 | 0.2 | |  |
| Yap | 8292 | 52000^d^ | 0.56 | 29.1 | 9.0 | 1.6 | 0.5 | 0.1 | |  |
| **Sum of all trenches (>6500 m) with known POC flux** |  | **739079** |  | **970** |  |  | **15.6** | **0.9** | |  |
| **Global Hadal Zone (>6500 m)** |  | **800500** |  | **1050** |  |  | **16.9** | **1.0** | |  |
| **Global Hadal Zone (>6000 m)** |  | **3440000^e^** |  | **4500** |  |  | **72.7** | **4.4** | |  |

Sources

*a. Stewart and Jamieson^24^ and Jamieson^35^.*

*b. Recalculated from this study. The original data reported in Stewart and Jamieson^24^ and Jamieson^35^ were erroneous.*

*c. Calculated with a Hg:POC ratio of 16.1 μg Hg. g OC^-1^ as per the main text*

*d. Fujiwara et al. ^48^*

*e. Harris et al.^10^*
